# Transmission Dynamics of *Schistosoma haematobium* among School-Aged Children: A Cohort Study on Prevalence, Reinfection and Incidence after Mass Drug Administration in the White Nile State of Sudan

**DOI:** 10.3390/ijerph182111537

**Published:** 2021-11-02

**Authors:** Yan Jin, Young-Ha Lee, Seungman Cha, In-Uk Choi, Hassan Ahmed Hassan Ahmed Ismail, Mousab Siddig Elhag, Sung-Tae Hong

**Affiliations:** 1 Department of Microbiology, Dongguk University College of Medicine, Gyeongju 38067, Korea; jinyan1024@dongguk.ac.kr; 2 Department of Infection Biology and Department of Medical Science, Chungnam National University College of Medicine, Daejeon 35015, Korea; choi76@cnu.ac.kr; 3 Department of Global Development and Entrepreneurship, Graduate School of Global Development and Entrepreneurship, Handong Global University, Pohang 37554, Korea; 4 Department of Disease Control, London School of Hygiene & Tropical Medicine, London WC1E 7HT, UK; 5 Communicable and Non-Communicable Diseases Control Directorate, Federal Ministry of Health, P.O. Box 303, Khartoum 1111, Sudan; hassanhassoon@hotmail.com (H.A.H.A.I.); mousabsiddig@gmail.com (M.S.E.); 6 Department of Tropical Medicine and Parasitology, Seoul National University College of Medicine, Seoul 03080, Korea; hst@snu.ac.kr

**Keywords:** *Schistosoma haematobium*, reinfection rate, incidence, mass drug administration, Sudan

## Abstract

The reinfection rate of schistosomiasis after mass drug administration (MDA) has not been documented in Sudan. We aimed to explore the transmission dynamics of urogenital schistosomiasis after MDA, targeting school-aged children in the White Nile State of Sudan, assessing the prevalence, reinfection rate, and incidence. A single dose of praziquantel (40 mg/kg) was administered to 1951 students in five primary schools from January to February 2018 immediately after a baseline survey, and follow-up surveys were performed at 2 weeks and 6 months after treatment. We examined *Schistosoma haematobium* eggs by centrifugation methods. The overall reinfection rate at 6 months after treatment was 9.8% (95% confidence interval: 0.5–17.4%). By school, the reinfection rate was highest in the Al Hidaib school, whose prevalence was highest at baseline. The reinfection rate was significantly higher in high-infection areas than low-infection areas (*p* = 0.02). Of the prevalence at 6 months in high-infection areas, 41% of cases were due to reinfection. MDA interventions are decided upon and undertaken at the district level. A more targeted treatment strategy should be developed with a particular focus on tracking high-risk groups, even within a school or a community.

## 1. Introduction

Human schistosomiasis, a neglected tropical disease, is caused by trematodes of the genus Schistosoma. These species live in the veins around the urinary bladder or intestine, and eggs are released in the urine or stool of the host [1]. These worms require snails to develop into the transmission stage and then they infect people who come into contact with infested water. *S. haematobium* adult worms reproduce sexually in humans; their eggs are released in urine into fresh water, where they can hatch. Free-living miracidia subsequently infect a suitable freshwater snail intermediate host (*Bulinus truncatus*), within which asexual reproduction occurs. Cercariae are then released by the snail back into the water, where they complete the lifecycle by infecting humans [2,3,4,5]. Humans are exposed to the infection by contacting water while engaging in domestic, recreational, and occupational activities [3]. Preschool-aged children, school-aged children, and some groups of people, such as irrigation workers and fishermen, have been reported to be at higher risk of schistosomiasis infection [4]. The disease is also known to be more prevalent among those who live in poor conditions, particularly in terms of water and sanitation [4,5]. Human schistosomiasis can cause hepatomegaly, splenomegaly, anemia, kidney malfunction, and stunting growth in children, and could also lead to cognitive dysfunction [6,7,8].

In Africa, *Schistosoma haematobium* and *Schistosoma mansoni* are the predominant species causing urogenital and intestinal schistosomiasis, respectively [9,10]. It is estimated that at least 90% of those requiring treatment for schistosomiasis live in sub-Saharan Africa, and at least 211 million people required preventive chemotherapy in 2019 [11].

The World Health Organization (WHO) recommends that young children living in endemic areas should be considered for treatment with praziquantel with the standard dose of 40 mg/kg [11,12]. Mass drug administration (MDA) with praziquantel has been the main intervention for schistosomiasis control in many endemic countries, mainly due to its safety, low cost, high compliance, and efficacy [12,13,14].

The global strategy of mass chemotherapy was mainly developed to control prevalence in high-endemic countries [12,15]. Due to the Sudanese government’s policy priority on neglected tropical diseases (NTDs) and its cooperation with development partners, schistosomiasis prevalence has been dramatically decreased throughout the country in the past few decades [16,17]. The Federal Ministry of Health (FMOH), Sudan, is formulating a new NTD strategy and transitioning its strategy from control to elimination of schistosomiasis [18].

The FMOH has implemented MDA against schistosomiasis every year, targeting school-aged children in the White Nile State in collaboration with the Korea International Cooperation Agency (KOICA) since 2009 [16,17,19,20]. The schistosomiasis prevalence was 28.5% in 2009 but decreased to 3.6% in 2019 [16,17,19,20]. Although water and sanitation improvements were conducted along the MDA, those interventions were restricted to a small portion of villages in the White Nile State. However, despite ongoing MDA interventions, high prevalence has been seen in some parts of the White Nile State until recently [16,17,19,20].

The FMOH is making every effort to develop integrated interventions to interrupt the transmission of schistosomiasis [18]. It is necessary to identify various parameters related to schistosomiasis transmission so that the FMOH can develop an informed policy and strategy to transition from the control of schistosomiasis towards elimination [21,22,23,24,25,26,27]. This prompted us to investigate changes in the prevalence, reinfection rate, and incidence after MDA interventions.

According to the latest systematic review, 14 studies have explored reinfection after MDA. However, the authors concluded that the reinfection rate varies substantially depending on the study area [28]. The substantial variance in the reinfection rate in that review might be explained by differences in environmental conditions, such as the presence of permanent water bodies, human behavior including water contact, the post-MDA survey timing, and the frequency of MDA [28]. To our knowledge, the reinfection rate and incidence after MDA have never been documented in Sudan.

For this reason, we aimed to explore transmission dynamics of urogenital schistosomiasis after MDA, targeting school-aged children in the White Nile State of Sudan, assessing the prevalence, reinfection rate, and incidence. In this study, we used an operational definition for reinfection, as those that were positive for schistosomiasis at the baseline survey became negative at the 2-week follow-up and reverted to positive at the 6-month follow-up.

## 2. Materials and Methods

### 2.1. Study Area

**Figure 1:**
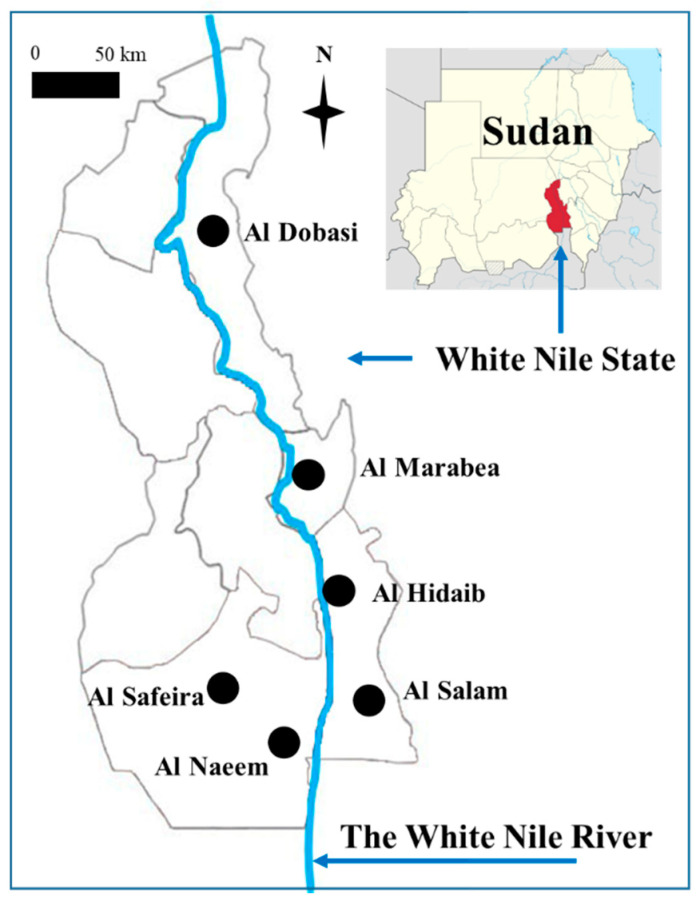
Study area (five primary schools in White Nile State, Sudan; blue line: the White Nile River).

We undertook this study from January to August 2018 at five primary schools in the White Nile State of Sudan (Figure 1). Based on the nationwide survey in 2017, we randomly selected schools in areas of relatively high prevalence.

The Blue and White Nile rivers meet in Sudan to form the Nile river. Among the countries that the Nile river crosses, Sudan has the widest river basin areas, and it also has large irrigated agricultural sectors along the banks of the Nile. The development of these irrigation schemes has led to significant environmental modification, favoring the spread of vector-borne diseases, including schistosomiasis [29]. Residents of these geographical environments along the Nile River have faced the risk of schistosomiasis for many centuries. During 1979–1990, the Blue Nile Health Project was implemented as a comprehensive plan to control malaria, schistosomiasis, and diarrheal diseases in the areas affected by the Gezira, Managil, and Rahad irrigation schemes. As a result, the prevalence of schistosomiasis was reduced from 53% to less than 10% [30]. However, there have been no further integrated programs to control schistosomiasis in Sudan thereafter.

The White Nile State is located along the White Nile river in the southeastern part of Sudan. The White Nile State is dominated by Muslims. The total population is 2.1 million. More than 30% of residents depend on farming and animal husbandry. The coverage of improved water and sanitation at the household level was 44% and 15%, respectively [16,20]. More than 33% of people reported practicing open defecation. Based on a nationwide survey, the statewide schistosomiasis prevalence of the White Nile State was 5.2%, ranging from 0.8% in the Kosti to 18.1% in the Al Gabalin locality. Three of the nine localities had prevalence of more than 5% [16]. Of the school-aged children, 33% reported routinely contacting infested water in their daily lives. Swimming, bathing, washing clothes and fetching water for domestic use were the main causes of water contact [20].

We previously investigated the annual patterns of snail populations that act as intermediate hosts of schistosomes, and monthly snail infection rates and ecological characteristics presumably related to snail populations. We collected snails for 1 year monthly at three different shore sites along the White Nile river in Sudan. Most of the collected snails were *Biomphalaria pfeifferi* and *Bulinus truncates* [31]. The population densities of snails and their infection rates varied across survey sites. The collected snails liberated *S. mansoni* and *S. haematobium* cercariae, as well as *Amphistome* and *Echinostome* cercariae. Infected snails were found from March to June. The ecological characteristics found to be associated with the absence of snails population were high turbidity, deep water, low vegetation coverage (near absence of vegetation), high water temperature, and high current speed [31]. The current of the White Nile river is slower than that of the Blue Nile river, providing favorable conditions for the intermediate host, snails, to survive [20]. According to a nationwide survey in 2017, the main type of schistosome in the White Nile river was *S. haematobium* [16]. The Korea Association of Health Promotion (KAHP), a non-governmental organization based in Korea, has implemented the intervention in collaboration with the State Ministry of White Nile.

### 2.2. Ethical Considerations

We obtained ethical approval from the institutional review board (IRB) of the KAHP (IRB approval no. 130750–20,164-HR-020) and also the Federal Ministry of Health, Sudan (FMOH/DGP/RD/TC/2016) before we started the survey. We collected samples from primary school students according to standard procedures recommended by the steering committee of the project composed of local government officials, WHO, and Sudanese and Korean parasitologists. Informed consent was obtained from the schoolteachers and students.

### 2.3. Sample Size Calculation

We used the following formula to estimate the sample size [24]:

$$\frac{\left(p1*q1+p2*q2\right)*{\left(Z_{\alpha /2}+2Z_{\beta }\right)}^{2}}{{\left(p1-p2\right)}^{2}}$$

$Z_{\alpha /2}$ is the value of alpha error, $Z_{\beta }$ is the value of beta error, *p*1 and *p2* are the prevalence before and after MDA, respectively, and *q*1 and *q*2 are the proportions of noninfection before and after MDA, respectively. Based on 5% alpha error and 80% study power, 6% pretreatment and 3% post-treatment prevalence, the sample size we estimated was 1260. Based on the substantial loss-to-follow-up in many existing studies, we estimated a 30% attrition rate, leading to an estimated sample of 1800 for this study.

### 2.4. Urine Sample Collection and Examination

Urine samples were collected for the baseline survey before Praziquantel treatment from all students of grade 1 through grade 7 in each school. We excluded students from the eighth grade because they would graduate during the study period. A single dose of Praziquantel (40 mg/kg) was administered to every child from January to February 2018 immediately after the baseline survey, and then follow-up surveys were performed at 2 weeks and 6 months after treatment. We examined *S. haematobium* eggs using the centrifugation method. Containers were delivered to the school in the early morning, and then collected on the same day. The collected samples were transferred to the Sudan–Korea Schistosomiasis Control Center in Kosti, White Nile State within 6 h.

After collection, parasitological examinations were carried out on the same day by laboratory technicians with more than 5 years of experience in urine and stool microscopy. To detect *S. haematobium* eggs, urine samples were subjected to centrifugation, as described elsewhere [19,32]. Specifically, 10 mL of urine in each tube was centrifuged at 1500 rpm for 5 min at room temperature, and the sediment was transferred onto 2–4 glass slides to examine the whole pellet. The slides were examined via microscopy to detect and count *S. haematobium* eggs. To estimate infection intensity, we calculated the number of eggs per individual 10 mL urine sample [32,33]. The mean intensity of *S. haematobium* infection at group level was expressed as the geometric mean intensity (GMI; number of *S. haematobium* eggs per 10 mL of urine: EP10).

For quality control, 10% of slides were randomly selected and re-examined at the end of each day by parasitology experts who were blinded to the results of the first examination. In case of disagreement, the results were discussed with the responsible technician and the discordant slides were re-examined until an agreement was reached.

### 2.5. MDA with Praziquantel

For the Praziquantel treatment, the State Ministry followed the schistosomiasis control protocol of FMOH, which was a modified version of WHO guidance [16]. If the prevalence is above 1% in a school-based study, all the students in the surveyed school should be provided with Praziquantel, and if it is above 3%, it should be provided to all students in every school of the district. Since the prevalence in the five target schools in this study was above 1%, all the students received Praziquantel after the baseline survey regardless of their infection status. The teachers and project team staff observed children swallowing the tablet when they provided Praziquantel to increase the compliance rate. Drugs were delivered at school by health workers, all of whom were White Nile Ministry of Health officials. They visited schools in the morning and administered Praziquantel to school children. The WHO dose pole was used to determine the dosage of Praziquantel (40 mg/kg) [12]. The WHO dose pole was used to determine the dosage (40 mg/kg) of Praziquantel (Distocide^®^, GMC, Khartoum, Sudan) [16].

### 2.6. Study Participants and Flow Diagram of the Cohort Study

**Figure 2:**
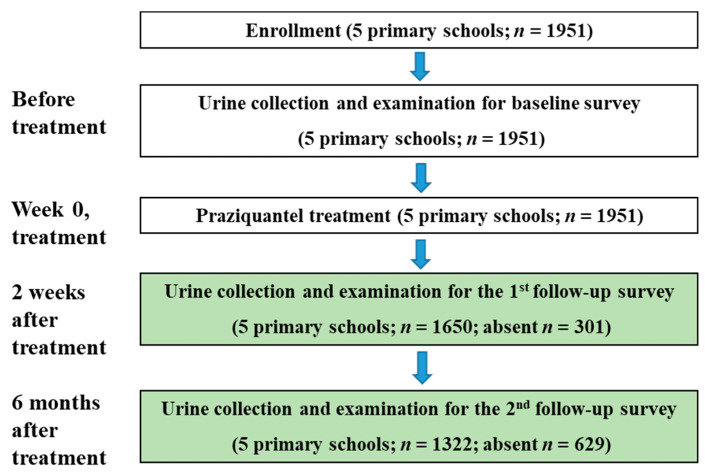
Flow chart of the longitudinal cohort study.

The general characteristics of the study population at baseline and follow-up are described in Table 1. The mean age of the enrolled children was 8.6 (±1.9) years. The flow diagram of the cohort study is illustrated in Figure 2.

### 2.7. Data Management and Statistical Analysis

We assessed the prevalence, infection intensity, reinfection rate, and incidence rate of *S. haematobium* infection over 6 months after Praziquantel treatment (Figure 2). Reinfection cases were defined as those that were positive for schistosomiasis at the baseline survey and became negative at the 2-week follow-up, and reverted to positive at the 6-month follow-up. Incidence was defined as the occurrence of new cases of infection at the 6-month follow-up survey among those who tested negative at the baseline survey. We compared the reinfection and incidence rates after categorizing the schools into two groups based on a baseline prevalence: high-infection areas (Al Hidaib primary school) and low-infection areas (all the other primary schools). We categorized the Al Hidaib school as a high-infection area and the others as low-infection area since the prevalence in the Al Hidaib school was considerably higher (above 40%) than that in the other schools (lower than 20%). To compare reinfection between the high- and low-infection areas, we used the Pearson chi-square tests of proportions. Due to deviation from a normal distribution, mean infection intensity of *S. haematobium* at the group level was calculated as geometric mean intensity (GMI) with units of EP10. For the relative risk of prevalence and mean difference of infection intensity of schistosomiasis after the treatment, multilevel mixed linear regression was run with age and sex as fixed factors and school as a random factor. All the statistical analyses were performed on the basis of a 5% alpha error. R statistics (R.4.1.0) and STATA 16 (StataCorp, College Station, TX, USA) were used to analyze the data. As supplementary analyses, we investigated the cure rate (CR) and egg reduction rate (ERR). For the CR, we calculated the proportion of children who were positive for schistosomiasis and became negative after treatment. For the ERR, we calculated the percentage reduction in the geometric mean egg count of *S. haematobium* only in positive students after treatment compared to the baseline value.

## 3. Results

At baseline, 269 of 1951 (13.8%) children were positive for *S. haematobium*. The baseline prevalence was slightly higher in boys than in girls (14.4% and 12.6%, respectively). The baseline prevalence of *S. haematobium* infection varied remarkably by school; it was highest in the Al Hidaib primary school (41.1%) and lowest in Al Sefeira (3.3%) (Table 2). Prevalence and intensity were different by school throughout the three rounds of the survey, except for intensity at 2 weeks after treatment (Table 2 and Table 3). A significant difference in prevalence by age was consistently found throughout the study period (Figure S1). At baseline, younger children had higher prevalence, but instead had a lower prevalence than older children at 2 weeks and 6 months after Praziquantel treatment. Infection intensity was significantly different by age only at baseline, where it peaked among children who were 9 years of age (Table S1). The pretreatment prevalence of heavy infection (≥50 eggs/10 mL of urine) was 21.6%, which was significantly higher than that of the other schools (16.4%, *p* < 0.001).

The overall prevalence decreased to 6.6% at 2 weeks and 4.2% at 6 months after the MDA intervention. The relative risk of schistosomiasis prevalence compared to the baseline value was 0.50 at 2 weeks after MDA (95% confidence interval (CI): 0.26–0.97; *p* = 0.04) and 0.33 at 6 month (95% CI: 0.18–0.58; *p* < 0.001). The geometric mean intensity of *S. haematobium* infection decreased to 4.5 EP10 at 2 weeks after treatment from 13.7 EP10 at baseline (arithmetic mean difference (MD) between the values of baseline and at two weeks = −12.6, 95% CI: −21.5 to −3.7; *p* = 0.006) The mean difference at 6 months was −16.1 EP10 (95% CI: −28.0 to −4.2; *p* = 0.008) (Table 3). By school, the highest baseline GMI values were seen in Al Naeem (19.8 EP10) and Al Hidaib (18.4 EP10).

The overall CR was 82.6% and 89.6% and the ERR was 67.2% and 55.5% at 2 weeks and 6 months after MDA, respectively (Table 4). Among those who were positive for *Schistosoma haematobium*, the follow-up rate was 77.0% and 61.0% at 2 weeks and 6 months after MDA. The ERRs were not significantly different at both 2 weeks (*p* = 0.19) and 6 months (*p* = 0.10) after Praziquantel treatment between schools. The CRs were not significantly different between schools at 2 weeks (*p* = 0.53), whereas they showed a significant difference at 6 months after Praziquantel treatment (*p* = 0.01).

The overall reinfection rate at 6 months after treatment was 9.8% (95% CI: 0.5%, 17.4%). By school, the reinfection rate was highest in the Al Hidaib school, which had the highest prevalence at baseline (Table 5). The reinfection rate was significantly higher in high-infection areas than in low-infection areas (*p* = 0.02). There were no cases of reinfection in Al Dobasi, Al Salam, and Al Naeem primary schools. Of the prevalence at 6 months after MDA, 41% of cases were due to reinfection.

**Figure 3:**
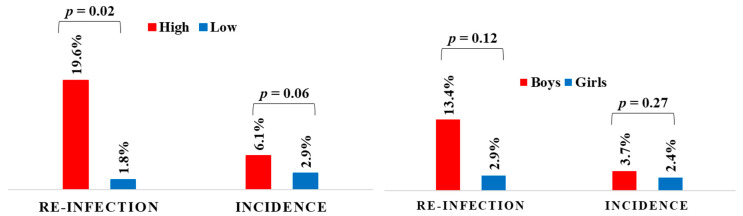
Reinfection rate and incidence in high- and low-infection areas (**left**) and among boys and girls (**right**).

Boys tended to have a higher reinfection rate than girls, but the difference was not statistically significant (*p* = 0.12) (Figure 3).

Of 1158 negative children in the baseline survey, 38 children were newly infected 6 months after the treatment, corresponding to an overall incidence of 3.3%. The incidence rates of boys and girls at 6 months after treatment were 3.7% and 2.4%, respectively.

## 4. Discussion

This is the first longitudinal cohort study to examine reinfection and incidence after MDA with a single dose of praziquantel (40 mg/kg) in Sudan. We found that the reinfection rate was 9.8%, which was lower than the mean value of the latest review (17.6%) but higher than the rate (1.2%) in Zimbabwe [34]. The previous remarkable achievement in terms of the lowest reinfection rate reported in Zimbabwe was made possible because MDA interventions were applied every 8 weeks for 2 years, which would not be practically feasible in Sudan due to financial limitations.

We compared the reinfection rate between high- and low-infection areas after categorizing the Al Hidaib primary school as a high-infection area and the others as low-infection areas; the pretreatment prevalence in these areas was 41.1% and 9.1%, respectively.

We found that the reinfection rate was 13 times higher in the high-infection area than in the other areas. More specifically, reinfections did not take place in three out of the five schools. Most cases of reinfection were detected in the Al Hidaib school. The 19.6% reinfection rate in the high-infection area was alarming, considering that these individuals were reinfected within just a 6-month period after praziquantel treatment, although the prevalence at 6 months (10.3%) did not revert to the pretreatment level.

Mass drug administration is a representative strategy to control schistosomiasis in endemic countries. However, persistent hotspots (i.e., areas with a high schistosomiasis burden despite repeated MDA) have been reported in many countries [18,19,20,21]. Insufficient coverage and reinfections among those treated were reported as limitations of MDA [21,22]. Despite the relatively high efficacy of praziquantel, reinfections took place rapidly and the prevalence after praziquantel treatment could reach the pretreatment level [15,31,35].

For this reason, we believe that prevalence should not be used as the only result-management indicator for schistosomiasis-related projects, even those solely involving MDA interventions. Otherwise, we might have been unduly satisfied with the reduced prevalence after MDA interventions, not knowing that a substantial proportion of children had been reinfected in such a short time period. Not considering multiple parameters relating to transmission dynamics would make it difficult to formulate adequate intervention strategies, particularly in areas where regular MDA interventions are already taking place, such as the study area (White Nile State, Sudan) [21,26]. It is worth noting that the maximum follow-up was 6 months in this study, whereas MDA was carried out only once per year in the study area; therefore, the reinfection rate in this study might have been underestimated and more children would have been reinfected up until the following MDA intervention. Another reason for applying caution when interpreting the reinfection rate lies in the high attrition rate in this cohort study. A considerable number of enrolled children were lost to the follow-up examination, although the attrition rate (32%) was lower than in some previous studies [35,36,37]. Absence of the enrolled students at school at the time of the follow-up survey was the only reason for loss to follow-up. We infer that the students we lost might have had different characteristics in many aspects, including their households’ socioeconomic characteristics and their behaviors, although we could not collect those data. Those students might have been absent because they had to do household chores, which could encompass fetching water, doing laundry, and watering livestock using infested water [16]. In this regard, there might have been a systematic difference between the students we followed and lost, which might have led to an underestimation of the reinfection rate.

Similarly, the considerable decrease in prevalence in Al Salam school at 6 months compared to 2 weeks after treatment might have been due to a substantial rate of loss to follow-up. More than half of the enrolled students were lost to follow-up in this school, and those who were absent at school at the follow-up survey might have had different characteristics from the other students. If the students were absent from school because of household chores, swimming, or playing in water bodies, the prevalence might have been underestimated at in the follow-up survey. Caution is needed when interpreting the significant difference in the CRs at 6 months of follow-up between schools because the rate of loss to follow-up was substantial and varied by school.

The high reinfection rate in the Al Hidaib primary school might be explained by levels of exposure in this area. This school is located near the White Nile river, unlike the four other schools (Figure 1). Thus, children in this area might experience high transmission due to their frequent exposure to infested water. This is consistent with previous research highlighting proximity to water bodies as a characteristic of persistent hotspots [21]. Similarly, the high prevalence of heavy infections might partially explain the high reinfection rate in the Al Hidaib school. This finding is consistent with a previous study, according to which the pretreatment prevalence of heavy infections was associated with the prevalence after praziquantel treatment [20].

The protective effect of acquired immunity to schistosome infection in high-transmission areas suggested by some studies was not detected in this study [38,39,40]. It is also possible that the apparent lack of acquired immunity in high-infection areas was primarily due to excessively intense transmission related to higher exposure, masking resistance to reinfection.

The reinfection rate was higher in boys than in girls, but the difference was not statistically significant (*p* = 0.06). The nonsignificance of the statistical test might have been partly due to the small sample size. The existing literature on differences in the reinfection rate by sex shows mixed results [28,41]. In our previous research in Sudan, a higher prevalence was found among boys than among girls [16]. The tendency for higher prevalence among boys was associated with their higher frequency of risky behaviors involving contact with infested water.

Another important finding is that about half of the children (909 school-aged children) were free from *S. haematobium* infection for 6 months, meaning that they remained negative during the entire survey period. This shows that the infection was concentrated in high-risk groups. More than 40% of those who were positive for schistosomiasis at 6 months were reinfected children. The higher prevalence after the treatment in high-infection areas compared to the low-infection areas is largely because of reinfected people. All in all, this points to the argument that there were high-risk groups even among students within a school.

It must be more effective when the elimination programs incorporate integrated interventions tailored for high-risk groups in endemic areas, such as more frequent MDA interventions targeting high-prevalence communities or populations, sustainable health education, and water and sanitation (WASH) improvement at the community, school, and household levels.

As a supplementary analysis, we examined the cure rate and egg reduction rate in this study after MDA treatment. Compared to previous studies, the cure rate was higher, and the egg reduction rate was lower [42]. This might have been in part because of the high compliance rate and low baseline value of egg counts [42,43].

Caution is needed when comparing our findings to those of previous studies. The previous studies reported in the latest review used different definitions of reinfection [28]. In some cases, the reinfection rates were either incidence or prevalence [35,38]. In this study, reinfections were defined as cases that were positive for schistosomiasis at the baseline survey and became negative at the 2-week follow-up, and reverted to positive at the 6-month follow-up.

This study has several limitations. First, the attrition rate was high, as stated above. A community-based survey would be appropriate to investigate the reinfection rate, as it would be possible to track out-of-school children and students absent from school, although seasonal migration should also be taken into account in that case. We believe that a community-based survey could be a complementary method rather than a replacement of a school-based survey.

Second, the urine examinations in this study were performed by a single egg screening process. Examining two or more specimens at different time points would likely reduce the variability of the serial cohort results of each participant because eggs are not passed regularly. The results based on a single screening would over- or underestimate the true rate of infection. According to the recent study on the population dynamics of snails in the White Nile river, the density of snails varied substantially from month to month, peaking in April and May, and snails were not present from August through October [31]. This suggests the possibility of high seasonal variation in reinfection and incidence, which was not taken into consideration in this study. Distance to infested water bodies, the presence of improved water and sanitation at the household, school, and community levels, open defecation, snail density, demographic characteristics, and socioeconomic status could have strong direct or indirect associations with a high risk of transmission [28]. However, these variables were not investigated in this study. A future study accounting for out-of-school children, absent students, seasonal variation in transmission of infection, and key characteristics of persistent hotspots should be warranted.

Praziquantel kills only adult worms and does not clear the entire infection in a host. Juvenile worms may develop in a host after MDA treatment, and this should not be considered as reinfection. Since praziquantel kills only adult worms, we could therefore infer that juvenile worms may survive after praziquantel treatment and become adult worms. Such cases are not reinfections. However, we could not take this possibility into consideration, which is another limitation of this study. Previous studies examining the efficacy of praziquantel used periods of 3 weeks to 3 months after treatment, and thus the 2-week period after treatment in this study might have been relatively short [42]. Still, we think that the issue of the follow-up period did not substantially affect the diagnostic results because more than 80% of the orally administered dose of praziquantel in humans is absorbed by the gastrointestinal tract, and it reaches its maximum plasma concentration within 1–2 h [44]. Praziquantel is excreted primarily through urine (60–80%) and bile and feces (15–35%) within 24 h [44]. The half-life of praziquantel is 1–2 h, and only a trace amount exists after 24 h [44].

## 5. Conclusions

Despite these concerted efforts, the prevalence in some areas has remained high. We detected that the resurgence of infection occurred in a large part because there were some high-risk sub-groups within a school who were repeatedly infected with schistosomiasis. MDA interventions are decided upon and undertaken at the district level according to the WHO guidelines. It is time to formulate a new MDA strategy highlighting more focalized interventions tailored for high-risk groups in high-transmission areas even within a school or community. More frequent treatments targeting high-risk groups are also strongly recommended.

## Figures and Tables

**Table 1:** General characteristics of the study population at baseline and follow-up rate.

School Name	Sample Size at Baseline	Mean Age (SD) in Years	Percentage of Boys (%)	Follow-up Rate (%)	
				2 Weeks	6 Months
Al Dobasi	383	8.7 (2.0)	100	90.1	73.9
Al Hidaib	285	8.8 (1.8)	42	71.6	74.7
Al Salam	434	8.6 (2.0)	45	75.3	49.5
Al Naeem	330	8.8 (2.0)	100	91.2	75.2
Al Sefeira	519	8.4 (1.8)	55	91.1	69.9
Total	1951	8.6 (1.9)	67	84.6	67.8

**Table 2:** The prevalence of *S. haematobium* at baseline, at 2 weeks, and at 6 months after mass drug administration.

		Baseline Survey			2-Week Follow-Up Survey						6-Month Follow-Up Survey					
		No. ^1^	(+)	%	No.	(+)	%	Relative Risk	95% CI	*p*-Value	No.	(+)	%	Relative Risk	95% CI	*p*-Value
School																
	Al Dobasi	383	25	6.5	345	7	2	0.29	0.14–0.61	0.001	283	19	6.7	1.04	0.49–1.81	0.90
	Al Hidaib	285	117	41.1	204	24	11.8	0.24	0.17–0.36	<0.001	213	22	10.3	0.26	0.18–0.38	<0.001
	Al Salam	434	69	15.9	388	61	15.7	0.97	0.71–1.33	0.87	215	6	2.8	0.17	0.08–0.40	<0.001
	Al Naeem	330	41	12.4	223	4	1.8	0.16	0.07–0.38	<0.001	248	2	0.8	0.06	0.01–0.27	<0.001
	Al Sefeira	519	17	3.3	490	13	2.7	0.81	0.41–1.63	0.56	362	6	1.7	0.52	0.22–1.23	0.13
Sex																
	Boys	1308	188	14.4	1079	62	5.7	0.48	0.29–0.79	0.004	897	43	4.88	0.44	0.26–0.77	0.004
	Girls	643	81	12.6	571	47	8.2	0.67	0.28–1.62	0.38	425	12	2.8	0.22	0.12–0.43	<0.001
Total		1951	269	13.8	1650	109	6.6	0.5	0.26–0.97	0.04	1322	55	4.2	0.33	0.18–0.58	<0.001

^1^ Number of examined school-aged children (SACs). (Prevalence was different by school throughout the 3 rounds of the survey).

**Table 3:** The geometric mean intensity (GMI) of *S. haematobium*-infected children.

Variables		Baseline Survey		2-Week Follow-Up Survey					6-Month Follow-Up Survey				
		Positive No. ^1^	GMI ^2^	Positive No.	GMI ^2^	Mean ^3^ Difference	95% CI	*p*-Value	Positive No.	GMI ^2^	Mean ^3^ Difference	95% CI	*p*-Value
School	Al Dobasi	25	7.2	7	4.7	−1.2	−11.5, 9.1	0.82	19	4.8	−4.4	−9.9,1.2	0.12
	Al Hidaib	117	18.4	24	7.2	−20.2	−41.3, 1.0	0.06	22	6.8	−26.1	−47.7, −4.5	0.02
	Al Salam	69	9.6	61	4.3	−7.1	−14.6, 0.4	0.06	6	6.3	−5.8	−26.2, 14.5	0.57
	Al Naeem	41	19.8	4	6.4	−15.1	−70.2, 40.0	0.59	2	15.2	−2.8	−79.3, 73.7	0.94
	Al Sefeira	17	8.4	13	2.2	−13	−26.6, 0.6	0.06	6	6.4	−7.9	−27.9, 12.2	0.44
Sex	Boys	188	13.8	62	4.9	−12.5	−25.4, 0.5	0.06	43	6.2	−18.5	−33.8, −3.2	0.02
	Girls	81	13.5	47	4	−12.9	−21.8, −4.1	0.004	12	5.8	−12.9	−29.0, 3.3	0.12
Total		269	13.7	109	4.5	−12.6	−21.5, −3.7	0.006	55	6.1	−16.1	−28.0, −4.2	0.008

^1^ Number of students positive for *S. haematobium.* ^2^ GMI (geometric mean intensity). Intensity: number of *S. haematobium* eggs per 10 mL of urine (EP10). ^3^ Arithmetic mean (intensity was different by school at baseline and 6 months after Praziquantel treatment).

**Table 4:** Cure rate and egg reduction rate at 2 weeks and at 6 months after mass drug administration.

	Baseline		At 2 Weeks						At 6 Months					
School	Positive No. ^1^	GMI ^2^	Negative No. ^3^	F-U ^4^	F-U Rate ^5^	GMI ^2^	Cure Rate	ERR ^6^	Negative No. ^3^	FU ^4^	F-U Rate ^5^	GMI ^2^	Cure Rate	ERR ^6^
Al Dobasi	25	7.2	20	25	100.0%	4.7	80.0%	34.7%	14	16	64.0%	4.8	87.5%	33.3%
Al Hidaib	117	18.4	69	86	73.5%	7.2	80.2%	60.9%	67	81	69.2%	6.8	82.7%	63.0%
Al Salam	69	9.6	48	59	85.5%	4.3	81.4%	55.2%	33	33	47.8%	6.3	100.0%	34.4%
Al Naeem	41	19.8	20	22	53.7%	6.4	90.9%	67.7%	24	24	58.5%	15.2	100.0%	23.2%
Al Sefeira	17	8.4	14	15	88.2%	2.2	93.3%	73.8%	9	10	58.8%	6.4	90.0%	23.8%
Sex														
Boys	188	13.8	115	140	74.5%	4.9	82.1%	64.5%	94	108	57.4%	6.2	87.0%	55.1%
Girls	81	13.5	56	67	82.7%	4	83.6%	70.4%	53	56	69.1%	5.8	94.6%	57.0%
Total	269	13.7	171	207	77.0%	4.5	82.6%	67.2%	147	164	61.0%	6.1	89.6%	55.5%

^1^ Number of students who were positive for *S. haematobium*; ^2^ GMI (geometric mean intensity; intensity: number of *S. haematobium* eggs per 10 mL of urine (EP10)); ^3^ number of students who were negative for *S. haematobium*; ^4^ number of students who were followed up among those who were positive at baseline; ^5^ the proportion of students who were followed up among those who were positive at baseline; ^6^ egg reduction rate.

**Table 5:** Reinfection rate and incidence at 6 months after MDA ^1^.

Categories ^2^	No. of Children by Sex			No. of Children by School				
	Total	Boys	Girls	Al Dobasi	Al Hidaib	Al Salam	Al Naeem	Al Sefeira
+ - ∙	102	67	35	13	46	23	11	9
+ - +	10	9	1	0	9	0	0	1
Reinfection rate (%)	9.8	13.4	2.9		19.6			11.1
95% CI (%)	(0.5–17.4)	(7.1–24.0)	(0.4–18.0)		(10.4–33.7)			(1.5–50.6)
- ∙ ∙	1158	789	369	267	132	182	224	353
- ∙ +	38	29	9	17	8	6	2	5
Incidence rate (%)	3.3	3.7	2.4	6.4	6.1	3.3	0.9	1.4
95% CI (%)	(2.4–4.5)	(2.6–5.2)	(1.3–4.6)	(4.0–10.0)	(3.0–11.7)	(1.5–7.2)	(0.2–3.5)	(0.6–3.4)

^1^ Excluding lost children at 6 months; ^2^ + - ∙ total children positive at baseline and negative at 2-week follow-up; + - + children positive at baseline, negative at 2-week follow-up, and positive at 6-month follow-up; - ∙ ∙ total children negative at baseline; - ∙ + children negative at baseline and positive at 6-month follow-up.

## Data Availability

Data will be shared upon request (mousabsiddig@gmail.com).

## Supplementary Materials

The following are available online at https://www.mdpi.com/article/10.3390/ijerph182111537/s1, Figure S1: Prevalence of *S. haematobium* by age, Table S1: Infection intensity of *S. haematobium*.

## References

[1] Gryseels B. (2012). Schistosomiasis. Infect. Dis. Clin. N. Am..

[2] Bustinduy A.L., Parraga I.M., Thomas C.L., Mungai P.L., Mutuku F., Muchiri E.M., Kitron U., King C.H. (2013). Impact of polyparasitic infections on anemia and undernutrition among Kenyan children living in a *Schistosoma haematobium*-endemic area. Am. J. Trop. Med. Hyg..

[3] King C.H., Dangerfield-Cha M. (2008). The unacknowledged impact of chronic schistosomiasis. Chronic Illn..

[4] Ezeamama A.E., Bustinduy A.L., Nkwata A.K., Martinez L., Pabalan N., Boivin M.J., King C.H. (2018). Cognitive deficits and educational loss in children with schistosome infection—A systematic review and meta-analysis. PLoS Negl. Trop. Dis..

[5] Friedman J.F., Kanzaria H.K., Acosta L.U.Z.P., Langdon G.C. (2005). Relationship between *Schistosoma japonicum* and nutritional status among children and young adults in Leyte, the Phillipines. Am. J. Trop. Med. Hyg..

[6] WHO (2006). Preventive Chemotherapy in Human Helminthiasis.

[7] World Health Organization (2018). Schistosomiasis and soil transmitted helminthiases: Numbers of people treated in 2017. Wkly. Epidemiol. Rec..

[8] Wiegand R.E., Mwinzi P.N.M., Montgomery S.P., Chan Y.L., Andiego K., Omedo M., Muchiri G., Ogutu M.O., Rawago F., Odiere M.R. (2017). A persistent hotspot of *Schistosoma mansoni* infection in a five-year randomized trial of praziquantel preventative chemotherapy strategies. J. Infect. Dis..

[9] Lewis F.A., Tucker M.S. (2014). Schistosomiasis. Adv. Exp. Med. Biol..

[10] Hotez P.J., Kamath A. (2009). Neglected tropical diseases in sub-saharan Africa: Review of their prevalence, distribution, and disease burden. PLoS Negl. Trop. Dis..

[11] World Health Organization (2021). Schistosomiasis: Key Facts. https://www.who.int/news-room/fact-sheets/detail/schistosomiasis.

[12] World Health Organization (2002). Prevention and Control of Schistosomiasis and Soil-Transmitted Helminthiaisis.

[13] Kabuyaya M., Chimbari M.J., Mukaratirwa S. (2018). Efficacy of praziquantel treatment regimens in pre-school and school aged children infected with schistosomiasis in sub-Saharan Africa: A systematic review. Infect. Dis. Poverty.

[14] Zwang J., Olliaro P. (2017). Efficacy and safety of praziquantel 40 mg/kg in preschool-aged and school-aged children: A meta-analysis. Parasit Vectors.

[15] Savioli L., Gabrielli A.F., Montresor A., Chitsulo L., Engels D. (2009). Schistosomiasis control in Africa: 8 years after World Health Assembly Resolution 54.19. Parasitology.

[16] Cha S., Elhag M.S., Lee Y., Cho D., Ismail H.A.H.A., Hong S. (2019). Epidemiological findings and policy implications from the nationwide schistosomiasis and intestinal helminthiasis survey in Sudan. Parasit Vectors.

[17] Cha S., Hong S.T., Lee Y.H., Lee K.H., Cho D.S., Lee J., Chai J.Y., Elhag M.S., Khaled S.G.A., Elnimeiri M.K.M. (2017). Nationwide cross-sectional survey of schistosomiasis and soil-transmitted helminthiasis in Sudan: Study protocol. BMC Public Health.

[18] Federal Ministry of Health (2021). A Neglected Tropical Diseases Strategy.

[19] Lee Y.H., Yeong H.G., Kong W.H., Lee S.H., Cho H.I., Nam H.S., Ismail H.A., Alla G.N., Oh C.H., Hong S.T. (2015). Reduction of urogenital schistosomiasis with an integrated control project in Sudan. PLoS Negl. Trop. Dis..

[20] Cha S., Hong S.T., Lee J.S., Jeong H.G., Kwon I.S., Saed A.A.W., Elhag M.S., Ismail H.A.H.A., Amin M., Lee Y.H. (2020). Comparison of the Change in the Prevalence and Intensity of *Schistosoma haematobium* Infection Between High and Low Prevalence Areas of White Nile State, Sudan. Korean J. Parasitol..

[21] Tchuem Tchuenté L.A., Rollinson D., Stothard J.R., Molyneux D. (2017). Moving from control to elimination of schistosomiasis in sub-Saharan Africa: Time to change and adapt strategies. Infect. Dis. Poverty.

[22] World Health Organization (2021). A Global Strategy on Water, Sanitation and Hygiene to Combat Neglected Tropical Diseases 2021–2030.

[23] World Health Organization (2021). Ending the Neglect to Attain the Sustainable Development Goals A Road Map for Neglected Tropical Diseases 2021–2030.

[24] Bizimana P., Ortu G., Van Geertruyden J.P., Nsabiyumva F., Nkeshimana A., Muhimpundu E., Polman K. (2019). Integration of schistosomiasis control activities within the primary health care system: A critical review. Parasit Vectors.

[25] Casulli A. (2021). New global targets for NTDs in the WHO roadmap 2021–2030. PLoS Negl. Trop. Dis..

[26] Lu X.T., Gu Q.Y., Limpanont Y., Song L.G., Wu Z.D., Okanurak K., Lv Z.Y. (2018). Snail-borne parasitic diseases: An update on global epidemiological distribution, transmission interruption and control methods. Infect. Dis. Poverty.

[27] Molyneux D.H., Savioli L., Engels D. (2017). Neglected tropical diseases: Progress towards addressing the chronic pandemic. Lancet.

[28] Zacharia A., Mushi V., Makene T. (2020). A systematic review and meta-analysis on the rate of human schistosomiasis reinfection. PLoS ONE.

[29] Steinmann P., Keiser J., Bos R., Tanner M., Utzinger J. (2006). Schistosomiasis and water resources development: Systematic review, meta-analysis, and estimates of people at risk. Lancet Infect. Dis..

[30] El Gaddal A.A. (1985). The Blue Nile Health Project: A comprehensive approach to the prevention and control of water associated diseases in irrigated schemes of the Sudan. J. Trop. Med. Hyg..

[31] Ismail H., Ahmed R.M., Lee Y.-H., Elhag M.S., Kim Y., Seungman C., Jin C. (2021). Population Dynamics of Intermediate-Host Snails in the White Nile River, Sudan: A Year-Round Observational Descriptive Study. Korean J. Parasitol.

[32] World Health Organization (1991). Sedimentation method. Basic Laboratory Methods in Medical Parasitology.

[33] Landouré A., Dembélé R., Goita S., Kané M., Tuinsma M., Sacko M., Toubali E., French M.D., Keita A.D., Fenwick A. (2012). Significantly Reduced Intensity of Infection but Persistent Prevalence of Schistosomiasis in a Highly Endemic Region in Mali after Repeated Treatment. PLoS Negl. Trop. Dis..

[34] Mduluza T., Ndhlovu P.D., Madziwa T.M., Midzi N., Zinyama R., Turner C.M., Chandiwana S.K., Nyazema N., Hagan P. (2001). The impact of repeated treatment with praziquantel of schistosomiasis in children under six years of age living in an endemic area for *Schistosoma haematobium* infection. Mem. Inst. Oswaldo Cruz.

[35] Mutsaka-Makuvaza M.J., Matsena-Zingoni Z., Tshuma C., Ray S., Zhou X.-N., Webster B., Midzi N. (2018). Reinfection of urogenital schistosomiasis in pre-school children in a highly endemic district in Northern Zimbabwe: A 12 months compliance study. Infect. Dis. Poverty.

[36] Kabuyaya M., Chimbari M.J., Manyangadze T., Mukaratirwa S. (2017). Efficacy of praziquantel on *Schistosoma haematobium* and re-infection rates among school-going children in the Ndumo area of uMkhanyakude district, KwaZulu-Natal, South Africa. Infect. Dis. Poverty.

[37] Houmsou R.S., Wama B.E., Agere H., Uniga J.A., Amuta E.U., Kela S.L. (2018). High Efficacy of Praziquantel in *Schistosoma haematobium*-Infected Children in Taraba State, Northeast Nigeria: A follow-up study. Sultan Qaboos Univ. Med. J..

[38] Mutapi F., Ndhlovu P.D., Hagan P., Woolhouse M.E.J. (1999). A comparison of re-infection rates with *Schistosoma haematobium* following chemotherapy in areas with high and low levels of infection. Parasite Immunol..

[39] Bashir M., Bickle Q., Bushara H., Cook L., Shi F., He D., Huggins M., Lin J., Malik K., Moloney A. (1994). Evaluation of defined antigen vaccines against *Schistosoma bovis* and *S. japonicum* in bovines. Trop. Geogr. Med..

[40] Yole D.S., Pemberton R., Reid G.D., Wilson A.R. (1996). Protective immunity to *Schistosoma mansoni* induced in the olive baboon *Papio anubis* by the irradiated cercaria vaccine. Parasitology.

[41] Mbanefo E.C., Huy N.T., Wadagni A.A., Eneanya C.I., Nwaorgu O., Hirayama K. (2014). Host determinants of reinfection with schistosomes in humans: A systematic review and meta-analysis. PLoS Negl. Trop. Dis..

[42] Olliaro P.L., Coulibaly J.T., Garba A., Halleux C., Keiser J., King C.H., N’Goran E.K., Raso G., Scherrer A.U., Figueiredo-Sousa J.C. (2020). Efficacy and safety of single-dose 40 mg/kg oral praziquantel in the treatment of schistosomiasis in preschool-age versus school-age children: An individual participant data meta-analysis. PLoS Negl. Trop. Dis..

[43] Farrell S.H., Truscott J.E., Anderson R.M. (2017). The importance of patient compliance in repeated rounds of mass drug administration (MDA) for the elimination of intestinal helminth transmission. Parasit Vectors.

[44] Chai J.Y. (2013). Praziquantel treatment in trematode and cestode infections: An update. Infect Chemother..

