# Supplementary material for: Transmission Dynamics of Schistosoma haematobium among School-Aged Children: A Cohort Study on Prevalence, Reinfection and Incidence after Mass Drug Administration in the White Nile State of Sudan
Source: Int J Environ Res Public Health. 2021 Nov 2;18(21):11537. doi: 10.3390/ijerph182111537 (PMC8583024; doi:10.3390/ijerph182111537)
Supplement: Supplementary file 1 [file ijerph-18-11537-s001.zip › ijerph-1348526-supplementary.pdf]

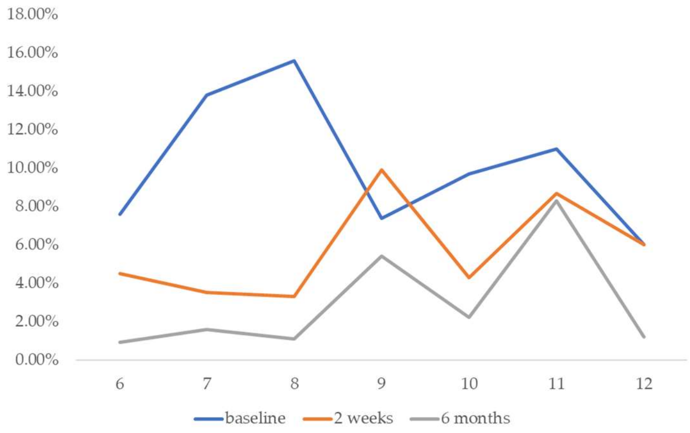

Figure S1. Prevalence of *S. haematobium* by age (baseline, 2 weeks and 6 months after PZQ treatment)

Table S1. Infection intensity of *S. haematobium* (number of eggs per individual 10 mL urine sample)

age	baseline	2 weeks	6 months
6	27.0(7.3)	8.1(3.0)	3.0(1.2)
7	34.2(5.2)	8.5(2.9)	9.5(4.4)
8	9.8(1.7)	3.6(0.9)	4.7(1.3)
9	73.4(14.3)	7.3(2.6)	9.2(1.9)
10	10.4(1.1)	14.0(9.3)	8.8(3.3)
11	23.1(2.8)	17.3(5.5)	12.5(4.3)
12	23.1(6.2)	21.6(10.1)	8.0(2.0)
	p<0.001	p=0.26	p=0.88
